# Synergistic Combination of Polymyxin B and Enrofloxacin Induced Metabolic Perturbations in Extensive Drug-Resistant *Pseudomonas aeruginosa*

**DOI:** 10.3389/fphar.2019.01146

**Published:** 2019-10-03

**Authors:** Yu-Wei Lin, Mei-Ling Han, Jinxin Zhao, Yan Zhu, Gauri Rao, Alan Forrest, Jiangning Song, Keith S. Kaye, Paul Hertzog, Anthony Purcell, Darren Creek, Qi Tony Zhou, Tony Velkov, Jian Li

**Affiliations:** ^1^Monash Biomedicine Discovery Institute, Infection and Immunity Program and Department of Microbiology, Monash University, Clayton, VIC, Australia; ^2^Division of Pharmacotherapy and Experimental Therapeutics, Eshelman School of Pharmacy, University of North Carolina, Chapel Hill, NC, United States; ^3^Monash Biomedicine Discovery Institute, Infection and Immunity Program and Department of Biochemistry and Molecular Biology, Monash University, Clayton, VIC, Australia; ^4^Division of Infectious Diseases, Department of Internal Medicine, University of Michigan Medical School, Ann Arbor, MI, United States; ^5^Centre for Innate Immunity and Infectious Diseases, Hudson Institute of Medical Research, Clayton, VIC, Australia; ^6^Department of Molecular and Translational Sciences, School of Clinical Sciences at Monash Health, Monash University, Clayton, VIC, Australia; ^7^Drug Delivery, Disposition and Dynamics, Monash Institute of Pharmaceutical Sciences, Monash University, Parkville, VIC, Australia; ^8^Department of Industrial and Physical Pharmacy, College of Pharmacy, Purdue University, West Lafayette, IN, United States; ^9^Department of Pharmacology and Therapeutics, The University of Melbourne, Melbourne, VIC, Australia

**Keywords:** polymyxin, extensive drug-resistant, *Pseudomonas aeruginosa*, enrofloxacin, metabolomics

## Abstract

Polymyxins are used as a last-resort class of antibiotics against multidrug-resistant (MDR) Gram-negative *Pseudomonas aeruginosa*. As polymyxin monotherapy is associated with potential development of resistance, combination therapy is highly recommended. This study investigated the mechanism underlying the synergistic killing of polymyxin B and enrofloxacin against extensive drug-resistant (XDR) *P. aeruginosa*. An XDR isolate *P. aeruginosa* 12196 was treated with clinically relevant concentrations of polymyxin B (2 mg/L) and enrofloxacin (1 mg/L) alone or in combination. Metabolome profiles were investigated from bacterial samples collected at 1-and 4-h posttreatment using liquid chromatography with tandem mass spectrometry (LC-MS/MS), and data were analyzed using univariate and multivariate statistics. Significantly perturbed metabolites (*q* < 0.05, fold change ≥ 2) were subjected to pathway analysis. The synergistic killing by polymyxin B–enrofloxacin combination was initially driven by polymyxin B as indicated by the perturbation of lipid metabolites at 1 h in particular. The killing was subsequently driven by enrofloxacin *via* the inhibition of DNA replication, resulting in the accumulation of nucleotides at 4 h. Furthermore, the combination uniquely altered levels of metabolites in energy metabolism and cell envelope biogenesis. Most importantly, the combination significantly minimized polymyxin resistance *via* the inhibition of lipid A modification pathway, which was most evident at 4 h. This is the first study to elucidate the synergistic mechanism of polymyxin B–enrofloxacin combination against XDR *P. aeruginosa*. The metabolomics approach taken in this study highlights its power to elucidate the mechanism of synergistic killing by antibiotic combinations at the systems level.

## Introduction

Extensive drug-resistant (XDR) *Pseudomonas aeruginosa* is a major burden to the global health-care system and has been highlighted by the World Health Organization as a priority pathogen with “Serious” threat to human health (Mcphee et al., 2006; Gales et al., 2011; World Health Organization, 2014). Due to the dry discovery pipeline, few novel classes of antibiotics will become available in the near future (Boucher et al., 2013). “Old” polymyxins (i.e., polymyxin B and colistin, also known as polymyxin E) are a last-line therapy that are increasingly used for life-threatening infections caused by XDR *P. aeruginosa* (Falagas et al., 2005; Li et al., 2006; Nation and Li, 2009; Yahav et al., 2012; Nation et al., 2014; Cheah et al., 2016a). Although polymyxins remain effective against XDR *P. aeruginosa*, recent pharmacokinetic/pharmacodynamic (PK/PD) studies suggest that polymyxin monotherapy is potentially associated with increased emergence of resistance (Tam et al., 2005; Cheah et al., 2015; Cheah et al., 2016b). Moreover, reports of infections caused by XDR *P. aeruginosa*, including polymyxin-resistant XDR isolates, are on the rise (Hsueh et al., 1998; Adams et al., 2009; Gales et al., 2012; Goli et al., 2016). In a recent *in vitro* PK/PD study, we demonstrated that polymyxin B in combination with enrofloxacin is highly effective against XDR *P. aeruginosa*, which is resistant to both, and significantly minimizes the emergence of polymyxin resistance (Lin et al., 2018). However, the underlying mechanism of the synergistic killing by this novel drug combination remains unknown.

Metabolomics investigates dynamic global metabolite levels in biological systems in response to biological stimuli or perturbations (Chen et al., 2007; Kaddurah-Daouk and Weinshilboum, 2014; Mastrangelo et al., 2014; Vincent et al., 2016). To date, metabolomics is increasingly employed in drug discovery and development to elucidate the mechanism of drug action (Peng et al., 2015). In the present study, we investigated the synergistic killing mechanism of polymyxin B and enrofloxacin combination against a clinical isolate of XDR *P. aeruginosa* using metabolomics. The mechanistic findings provide important pharmacological information for optimizing this promising combination in patients.

## Materials and Methods

### Chemicals and Reagents

A solution of polymyxin B (sulfate, Sigma-Aldrich, Castle Hill, NSW, Australia; batch number BCBD1065V) was freshly prepared in sterile Milli-Q water (Millipore Australia, North Ryde, NSW, Australia). Enrofloxacin (Sigma-Aldrich) was first dissolved in dimethyl sulfoxide (DMSO; Sigma-Aldrich) and subsequently diluted in sterile Milli-Q water to obtain a final DMSO concentration of ≤10% (v/v) (Tran et al., 2016a).

### Bacterial Strain and Culture

An XDR *P. aeruginosa* 12196 with a polymyxin MIC of 64 mg/L and enrofloxacin MIC of 4 mg/L was examined (Lin et al., 2018). *P. aeruginosa* 12196 was stored in tryptone soy broth with 20% glycerol at −80°C and sub-cultured onto nutrient agar plates before each experiment (Maifiah et al., 2016; Maifiah et al., 2017). Overnight culture was subsequently prepared in 10-ml cation-adjusted Mueller-Hinton broth (CAMHB) and diluted 100-fold using fresh media to prepare 200 ml of mid-logarithmic culture with a starting inoculum of approximately 10^8^ CFU/ml (Maifiah et al., 2016; Maifiah et al., 2017). To each bacterial culture, polymyxin B (2 mg/L), enrofloxacin (1 mg/L), or a combination was added. Bacterial culture without any antibiotics served as a control. Three biological replicates were prepared independently from different colonies of XDR *P. aeruginosa* 12196 on three consecutive days. Bacterial cultures were incubated at 37°C in a shaking incubator (180 rpm). Samples were collected at 0, 1, and 4 h and immediately quenched in a dry ice–ethanol bath for 30 s to halt the metabolism. Subsequently, the OD_600_ value of each sample was measured and normalized to 0.50 ± 0.02 with fresh CAMHB. Subsequently, 15 ml of each normalized sample culture was transferred to 15-ml Falcon tubes (Thermo Fisher Scientific, Melbourne, Australia) for metabolite extraction.

### Sample Preparation for Metabolomics Experiments

Metabolite sample preparation was carried out as reported previously (Han et al., 2018). Briefly, 15 ml of each bacterial culture was centrifuged at 3,200 × *g* at 4 C. The supernatant was discarded, and cell pellets were resuspended in cold 0.9% sodium chloride solution. Samples were centrifuged at 3,200 × *g* for 5 min to remove extracellular metabolites and media components. Following the washing step, bacterial pellets were resuspended in 0.5 ml of chloroform/methanol/water (1:3:1, v/v/v) containing 1 µM internal standards (CHAPS, CAPS, PIPES, and TRIS). Subsequently, bacterial samples were frozen in liquid nitrogen and thawed on ice to release intracellular metabolites. The samples were then centrifuged at 14,000 × *g* for 10 min, and 200 µL of supernatants was transferred into ultra-performance liquid chromatography (UPLC) vials for liquid chromatography with tandem mass spectrometry (LC-MS/MS) analysis. QC samples were prepared by mixing equal amounts of all tested samples and processed as a “real” sample outlined above.

### LC-MS/MS Analysis for Metabolomics

Metabolite samples were analyzed on a Q-exactive Orbitrap mass spectrometer coupled with a Dionex U3000 high-performance LC (HPLC; Thermo Fisher) with a ZIC-pHILIC column (5 µm, polymeric, 150 × 4.6 mm; SeQuant, Merck). The MS system was operated at 35,000 resolution in both positive and negative electrospray ionization modes with a detection range of 85–1,275 *m*/*z*. Column temperature was maintained at 25 C, and the mobile phase consisted of 20 mM of ammonium carbonate (solvent A) and acetonitrile (solvent B). Metabolites were eluted in a step gradient, starting with 80% solvent B at a flow rate of 0.3 ml/min followed by a linear gradient to 50% solvent B over 15 min (Maifiah et al., 2016; Maifiah et al., 2017). All samples were randomized and analyzed in a single LC-MS batch. Analytical reproducibility was monitored on the basis of pooled QC samples throughout the batch, which were periodically analyzed after groups of six samples. Analyses of a mixture of pure standards containing >250 metabolites were performed to assist in the identification of metabolites.

### Bioinformatics and Pathway Analyses

IDEOM (http://mzmatch.sourceforge.net/ideom.php) and mzMatch were employed for metabolomics analysis (Scheltema et al., 2011; Creek et al., 2012). Raw mass spectrometric data files were processed as described previously based on the intensity (>100,000 counts), shape (codadw > 0.8), and reproducibility (RSD < 0.5) of the LC-MS peaks. Elemental composition and exact mass were used for open-source database searching, including MassBank (http://www.massbank.jp). Putative metabolites were identified by accurate mass (±5 ppm) and retention time with authentic standards (<50%) as indicated by IDEOM confidence score of 9 or 10, or by accurate mass (±5 ppm) and predicted retention time to achieve an IDEOM confidence scores of ≥5 (Creek et al., 2012). Several different databases [e.g., *PseudoCyc*, Kyoto Encyclopedia of Genes and Genomes (KEGG), BioCyc HMDB, and LipidMaps] were used to map the metabolite, and global metabolomics profiles of samples were analyzed using univariate and multivariate analyses in MetaboAnalyst 4.0 (Xia et al., 2015). Data were filtered using interquartile range (IQR), normalized relative to the median, log_2_ transformed, and auto-scaled. PCA was performed to identify and remove outliers that were defined as samples outside of ±2 standard deviations (SDs) along the principal component 1 axis (PC1). Statistical significance of differences between metabolites was determined using one-way analysis of variance (ANOVA), Benjamini–Hochberg multiple testing correction (*q* < 0.05), Fisher’s least significant difference (LSD) test, and fold change (FC; log_2_FC ≥ 1). FC values were calculated using raw intensity and geometric mean of the biological replicates. BioCyc (Karp et al., 2005), iPath (Letunic et al., 2008), and the KEGG (Kanehisa and Goto, 2000) were employed for pathway analysis.

## Results

### Metabolomics Profiles of *Pseudomonas aeruginosa* Treated With Polymyxin B, Enrofloxacin, and the Combination

**Figure 1:**
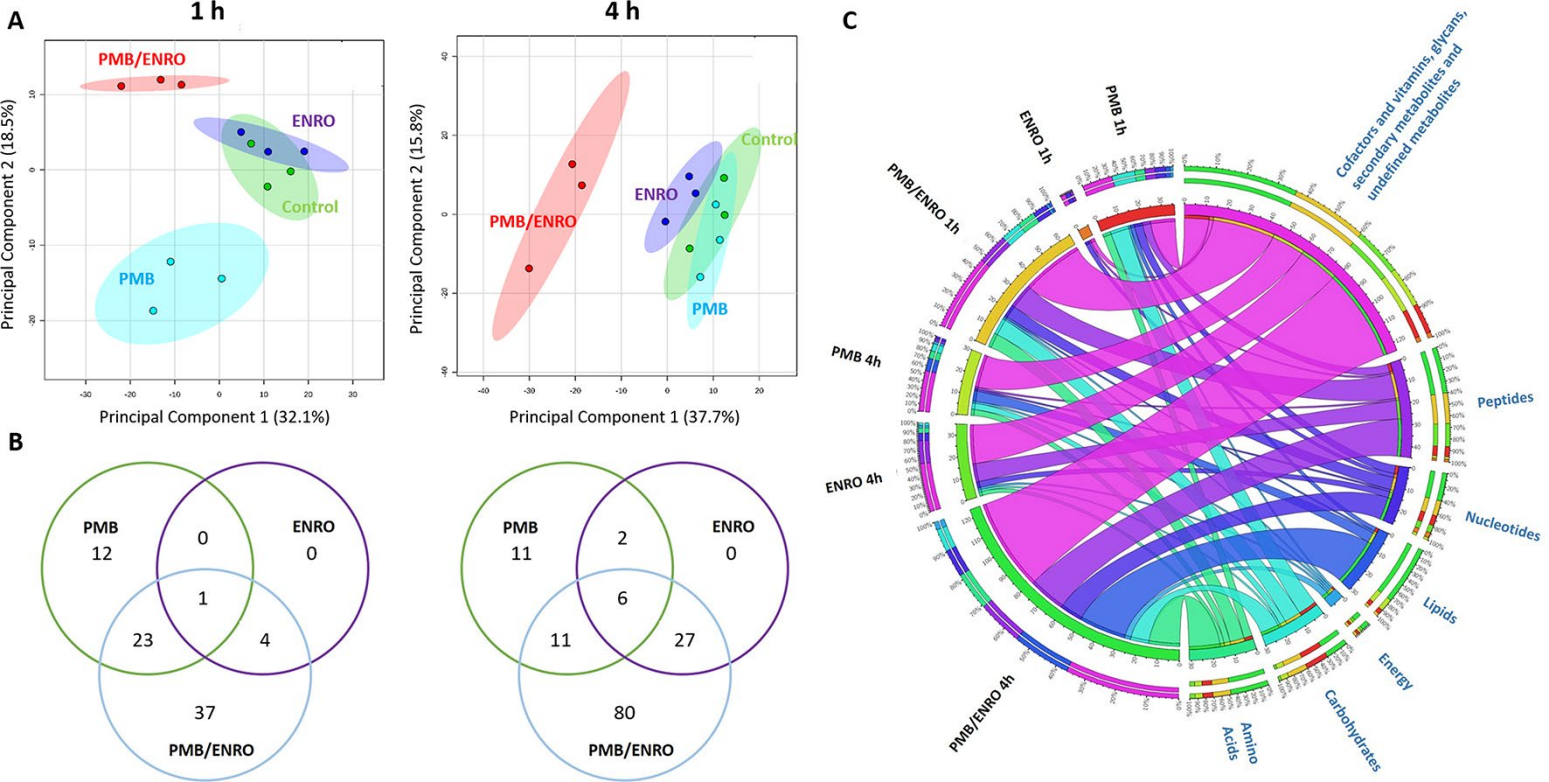
Metabolomics analyses of XDR *P. aeruginosa* 12196 treated with polymyxin B (PMB) and enrofloxacin (ENRO) alone or in combination (PMB/ENRO). **(A)** Principal component analysis (PCA) score plots for the metabolites from bacterial cultures treated with polymyxin B, enrofloxacin, and the combination at 1 and 4 h. Green, light blue, purple, and red represent untreated control, polymyxin B alone (PMB), enrofloxacin alone (ENRO), and the combination (COMB), respectively. **(B)** Venn diagram for the comparison of the numbers of metabolites that were significantly altered by each treatment at each time point. Significant metabolites were selected based on log_2_ fold change (FC) ≥ 1 and *q* < 0.05. **(C)** Bipartite graph connected different treatment groups with significantly altered metabolites in major classes.

**Figure 2:**
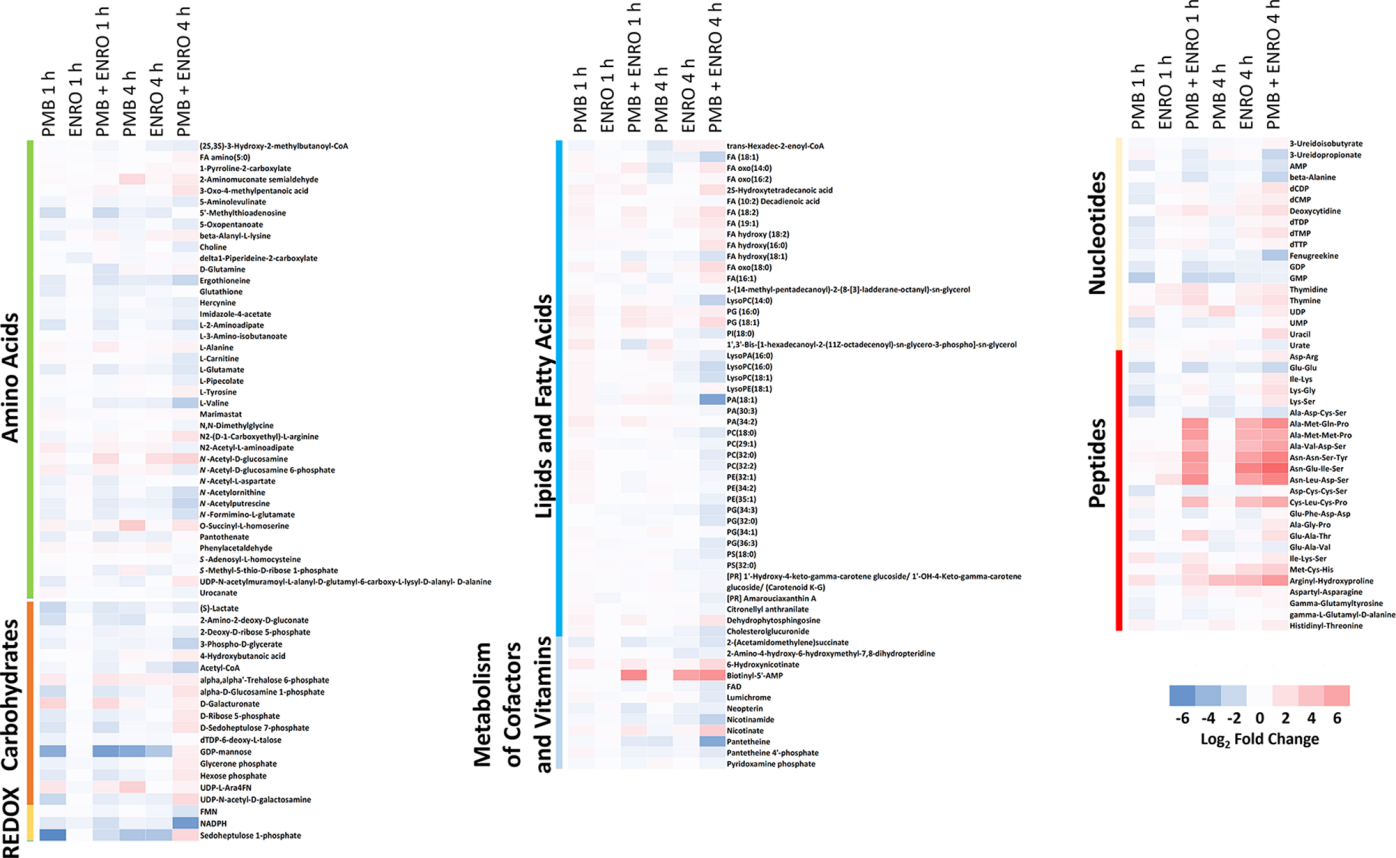
Heatmap profiles of the relative abundance of putative metabolites in XDR *P. aeruginosa* 12196 following treatment with polymyxin B alone (PMB), enrofloxacin alone (ENRO), and its combination (PMB + ENRO) at 1 and 4 h. Metabolites were grouped into different classes: amino acids, carbohydrates, energy, lipids (lipid metabolism, fatty acids, and phospholipids), co-factors and vitamins, nucleotides, and peptides. Only the significantly perturbed metabolites in the major perturbed pathways are included. Data represent log_2_FC compared with the untreated control samples at respective time points.

The intra-experimental variability was assessed based on the median relative standard deviations (RSDs) of the samples, which ranged from 15% to 24% (**Figure S1**) and were well within the acceptable limits for metabolomics studies (Kirwan et al., 2014). Furthermore, the principal component analysis (PCA) plots showed that all samples [including six quality control (QC) samples] were tightly clustered together, demonstrating an excellent reproducibility of our analytical methods (**Figure S1**). Univariate and multivariate analyses revealed that over 500 putative metabolites were identified in the metabolome of XDR *P. aeruginosa* 12196 induced by polymyxin B (2 mg/L) and enrofloxacin (1 mg/L) alone or in combination at 1- and 4-h postdrug treatment. The nature of these metabolites indicated that a wide range of pathways were perturbed (**Figures 1**, **2** and **S2**; **Supplementary Tables 1–2**). Univariate analysis showed that polymyxin B alone induced 6.3% (36) and 5.3% (30) metabolic changes at 1 and 4 h, respectively. Likewise, the combination induced 11.4% (65) and 21.8% (124) metabolic changes at 1 and 4 h, respectively. On the other hand, enrofloxacin alone induced minimal metabolic changes at 1 h (**Figures 1**, **2** and **S2**). PCA demonstrated that polymyxin B alone and in combination with enrofloxacin induced significant global metabolic changes as early as 1 h (**Figure 1A**). Perturbations in the metabolome of XDR P. aeruginosa 12196 induced by polymyxin B (2 mg/L) and enrofloxacin (1 mg/L) alone or in combination were evident at 1 and 4 h post drug treatment. Many metabolic features were shared between the two monotherapies and the combination with more significant changes at 4 h, demonstrating a time-dependent antibacterial effect by the drug combination (**Figure 1B**). At 1 h, the number of perturbed metabolites that were common between polymyxin B alone and the combination was much higher than that between enrofloxacin alone and the combination. Interestingly, at 4 h, the metabolic alterations were largely caused by the combination (**Figure 1B**). Overall, the combination of polymyxin B and enrofloxacin produced significantly greater perturbations in the metabolomes at 1 and 4 h than either polymyxin B or enrofloxacin alone.

Pathway analyses revealed that multiple metabolic pathways were affected by antibiotic treatments. In details, at 1 and 4 h polymyxin B alone significantly perturbed phospholipid and fatty acid metabolisms, whereas enrofloxacin alone had minimal metabolic perturbations on both metabolic pathways (**Figures 1**, **2** and **S2**). On the contrary, the combination therapy significantly perturbed a greater number of key metabolic pathways, including lipid, carbohydrate, nucleotide, and energy metabolism (**Figures 1**, **2** and **S2**). The levels of perturbed metabolites are provided in **Supplementary Tables 1–2**.

### Perturbations in Phospholipid and Fatty Acid Levels, and Lipid A Modification Pathway

**Figure 3:**
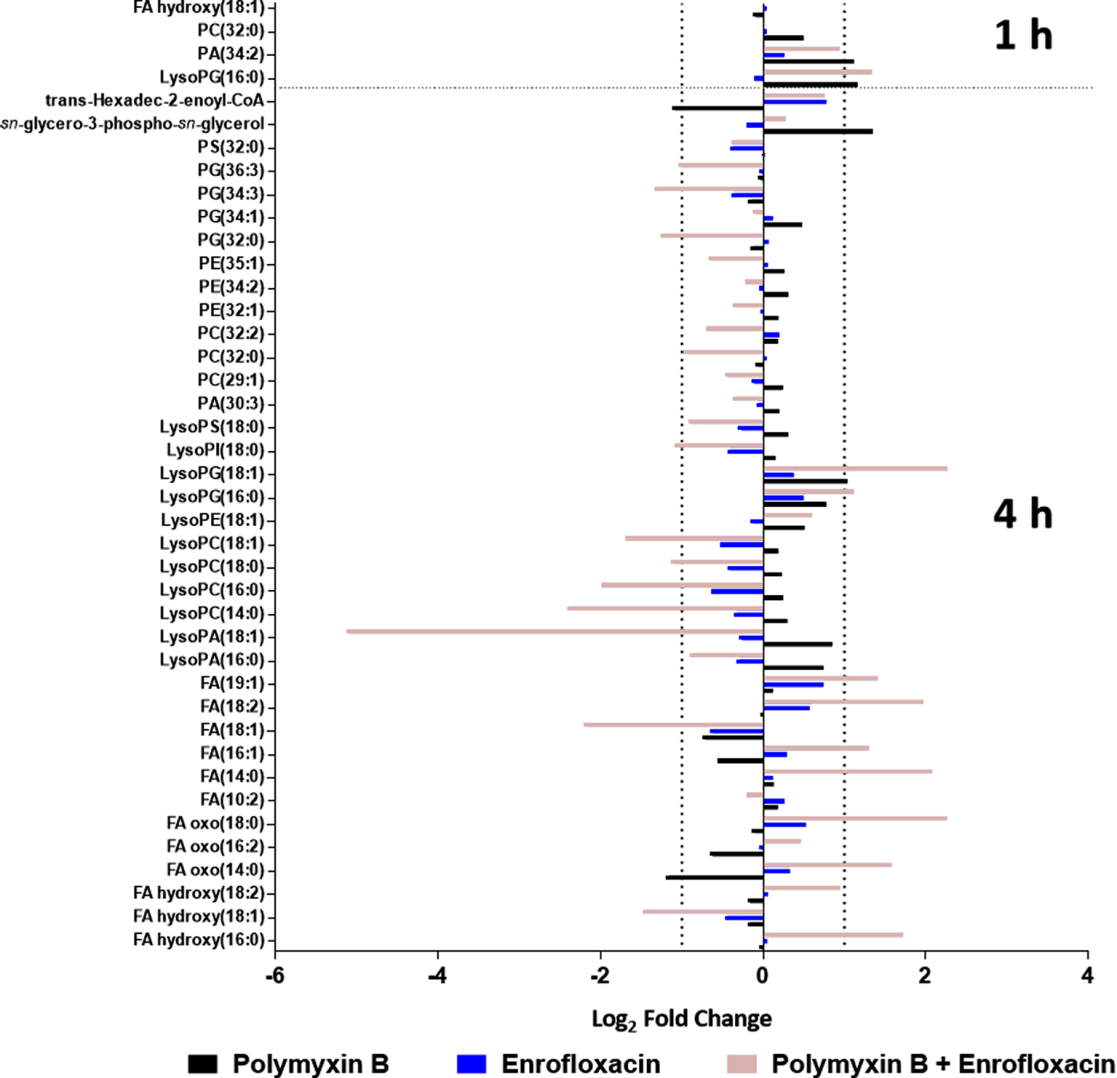
Metabolic perturbations in the phospholipid and fatty acid pathway following treatments with polymyxin B, enrofloxacin, and the combination against XDR *P. aeruginosa* 12196. Data represent log_2_FC compared with the untreated control samples at respective time points.

Polymyxin B alone and its combination with enrofloxacin significantly perturbed phospholipid and fatty acid levels at 1- and 4-h posttreatment (**Figures 2** and **3**). More specifically, at 4 h, the drug combination significantly decreased the levels of phospholipids, phosphatidylserine (PS), phosphatidylethanolamine (PE), and phosphatidylglycerol (PG) (**Figure 3**). Enrofloxacin alone did not have a significant impact on phospholipid levels. Interestingly, the decreased phospholipid levels were accompanied with an accumulation of a large number of fatty acids intracellularly at 4 h (**Figure 3**). Importantly, at 4 h, polymyxin B alone led to significantly increased levels of uridine 5′-diphospho-beta-(4-deoxy-4-formamido-l-arabinose) (UDP-l-Ara4FN) (log_2_FC = 2.95), a key precursor of 4-amino-4-deoxy-l-arabinose (l-Ara4N)-modified lipid A but not in the groups of enrofloxacin alone (log_2_FC = 0.11) or the combination (log_2_FC = 0.79) (**Figure 2**).

### Polymyxin B and Enrofloxacin Combination Significantly Altered Nucleotide Metabolism and Decreased Energy Metabolism

**Figure 4:**
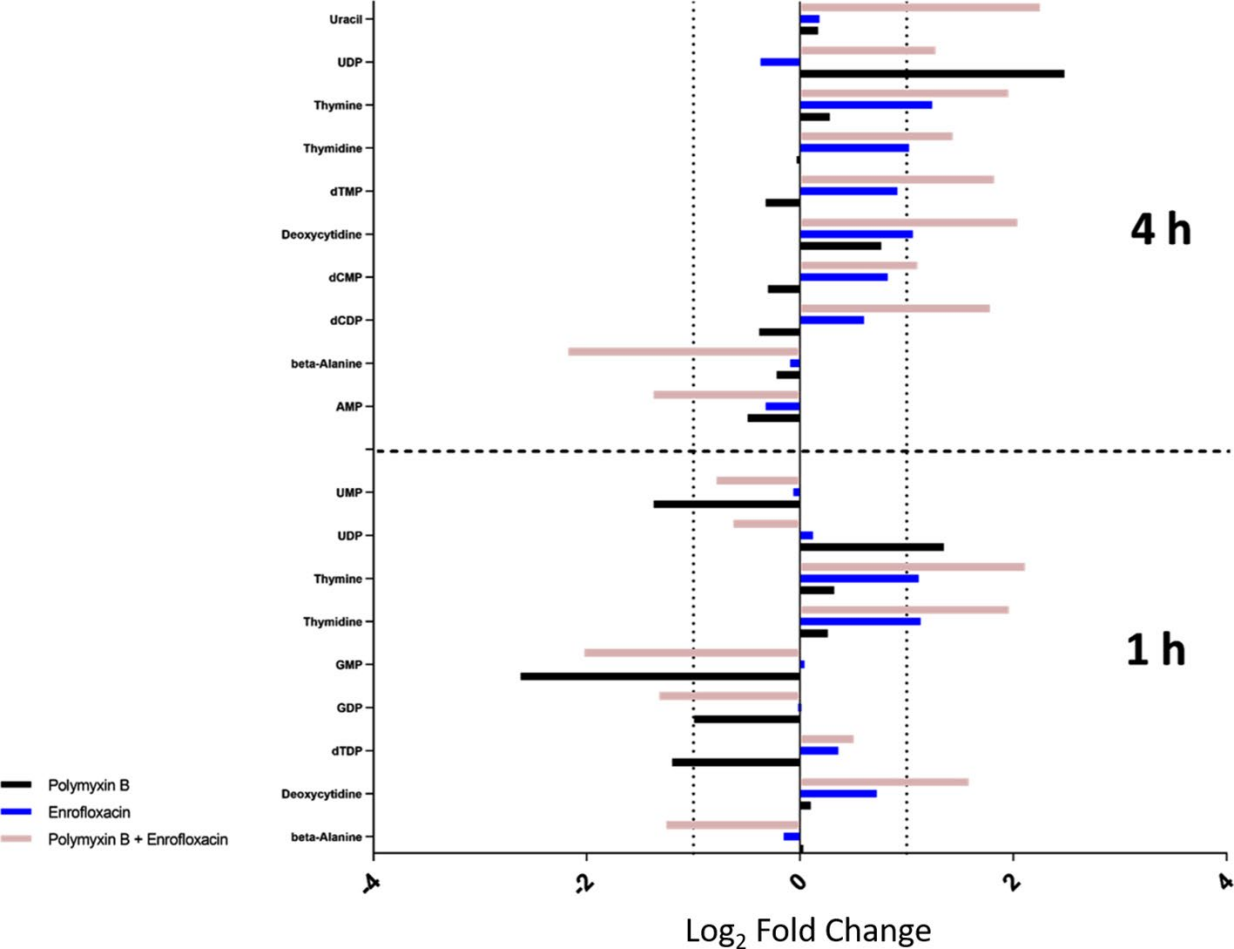
Metabolic perturbations in nucleotide metabolism at 1 and 4 h. Data represent log_2_FC relative to untreated control samples at respective time points.

Several intermediate metabolites in pyrimidine metabolism were significantly enriched at 4 h by the combination. In particular, the levels of deoxycytidine (log_2_FC = 2.04), deoxycytidine monophosphate (dCMP; log_2_FC = 1.10), deoxycytidine diphosphate (dCDP; log_2_FC = 1.78), thymine (log_2_FC = 1.95), thymidine (log_2_FC = 1.43), deoxythymidine monophosphate (dTMP; log_2_FC = 1.82), and uridine diphosphate (UDP; log_2_FC = 1.27) were all significantly increased at 4 h by the combination (**Figure 4**). For each monotherapy, only UDP (log_2_FC = 2.48) was elevated following polymyxin B alone at 4 h, while levels of deoxycytidine (log_2_FC = 1.06), thymine (log_2_FC = 1.24), and thymidine (log_2_FC = 1.02) were significantly increased by enrofloxacin alone at 4 h (**Figure 4**).

In contrast, the combination significantly decreased the levels of the metabolites in purine metabolism at 1 or 4 h. In details, the levels of guanosine monophosphate (GMP; log_2_FC = −2.02) and guanosine diphosphate (GDP; log_2_FC = −1.32) were significantly decreased by the combination at 1 h, whereas at 4 h, adenosine monophosphate (AMP; log_2_FC = −1.37) was significantly decreased (**Figure 4**). Polymyxin B alone also significantly perturbed GMP (log_2_FC = −2.62) at 1 h but had a minimal effect on purine nucleotide metabolism at 4 h (**Figures 4** and **S2**; **Supplementary Tables 1–2**). At the examined concentration, enrofloxacin alone had a minimal effect on purine metabolism at 1 and 4 h.

**Figure 5:**
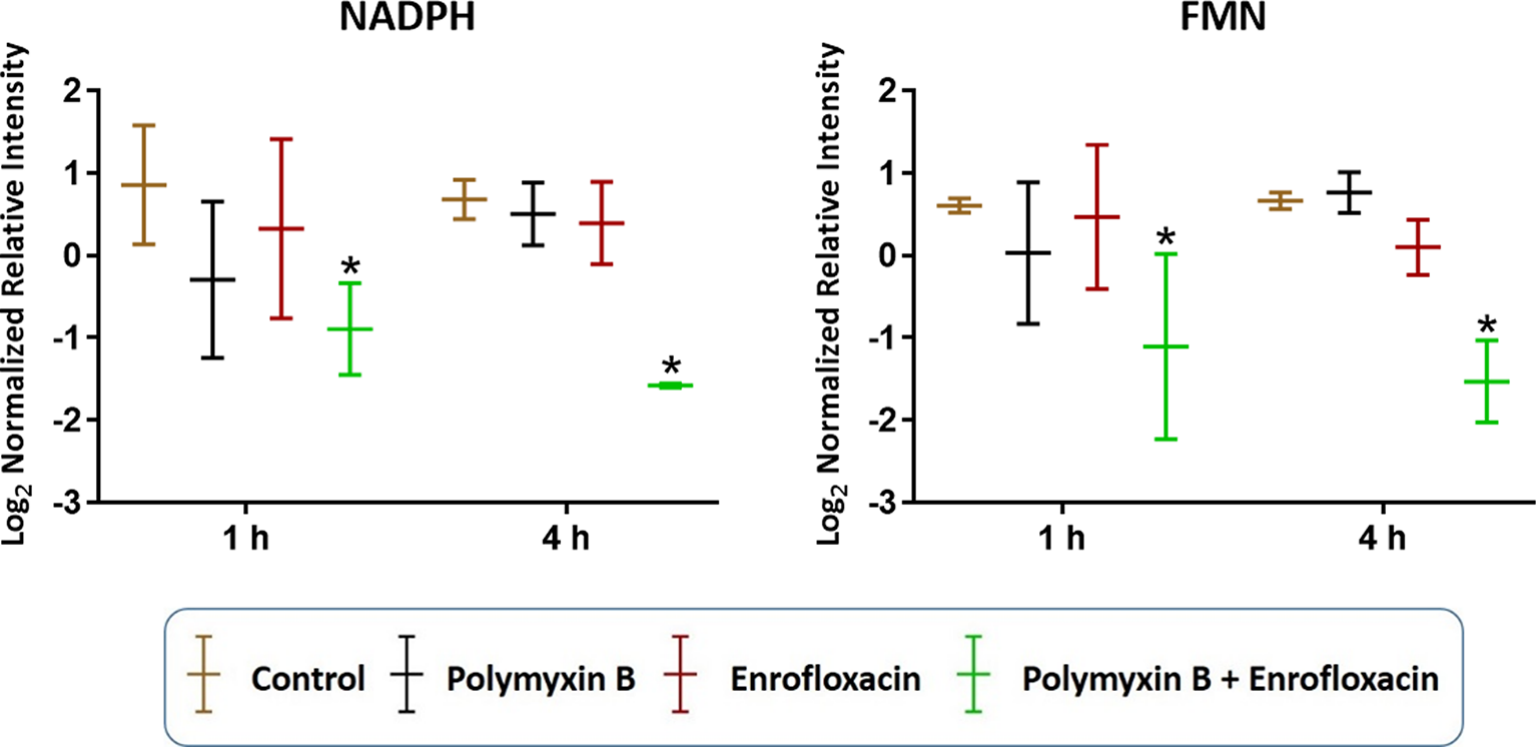
Depletion of key redox co-factors following treatments with polymyxin B, enrofloxacin, and the combination against XDR *P. aeruginosa* 12196. Bars labeled with an asterisk indicate significant changes in the abundance of metabolites (*q* < 0.05; ≥1 − log_2_FC). Data represent means of normalized intensity ± standard deviation (*n* = 3). Intensity was normalized relative to the median, log_2_ transformed, and auto-scaled.

In addition to nucleotide metabolism, metabolites related to energy metabolism were significantly depleted by the combination but not the monotherapies (except for sedoheptulose 1-phosphate). Specifically, at 1 and 4 h, two important redox co-factors, flavin mononucleotide (FMN; log_2_FC = −0.31 and −1.37, respectively) and nicotinamide adenine dinucleotide phosphate (NADPH; log_2_FC = −1.63 and −5.57, respectively), were significantly depleted by the combination (**Figure 5** and **Supplementary Tables 1–2**). Neither polymyxin B nor enrofloxacin alone exhibited significant effects on the levels of redox co-factors at 1 and 4 h.

### Polymyxin B and Enrofloxacin Combination Perturbed the Pentose Phosphate Pathway and Cell Envelop Biogenesis

**Figure 6:**
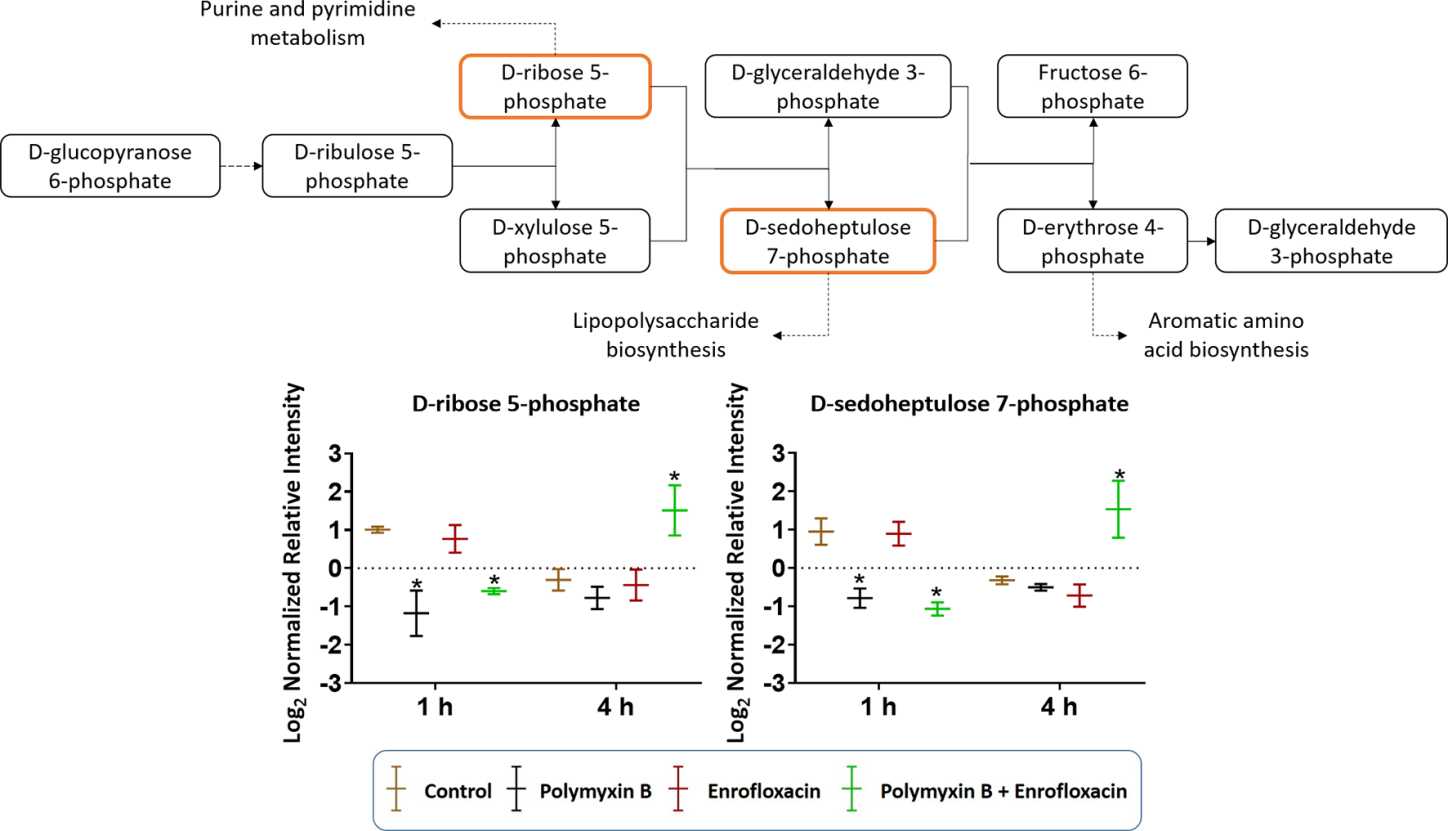
Metabolic perturbations in the pentose phosphate pathway in XDR *P. aeruginosa* 12196. Orange boxes indicate the metabolites that were significantly perturbed. Bar charts show the raw intensity at respective time points (1 and 4 h), and bars labelled with an asterisk indicate significant changes in the abundance of metabolites (*q* < 0.05; ≥1 − log_2_FC). Raw intensity was normalized relative to the median, log_2_ transformed, and auto-scaled. Data represent geometric means of normalized intensity ± standard deviation (*n* = 3).

The combination of polymyxin B and enrofloxacin significantly perturbed key intermediates in the pentose phosphate pathway (PPP) (**Figure 6**). Two key intermediate metabolites, d-ribose 5-phosphate (log_2_FC = −1.02 and −0.72, respectively) and d-sedoheptulose 7-phosphate (log_2_FC = −1.29 and −1.52, respectively), were significantly decreased at 1 h by polymyxin B alone and the combination. Interestingly, d-ribose 5-phosphate (log_2_FC = 1.47) and d-sedoheptulose 7-phosphate (log_2_FC = 1.75) were significantly enriched by the combination at 4 h (log_2_FC ≥ 1, *q* < 0.05) (**Figure 6**). Notably, neither glycolysis nor citric acid cycle was significantly perturbed by each monotherapy or the combination at the tested concentrations (**Figure S2** and **Supplementary Tables 1–2**).

**Figure 7:**
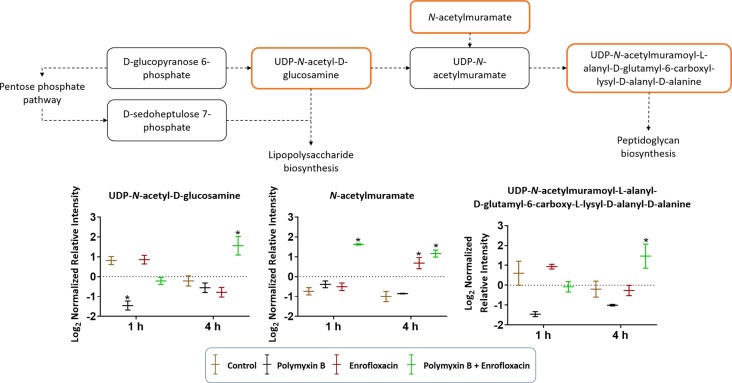
Metabolic perturbations in cell envelope biogenesis in XDR *P. aeruginosa* 12196. Orange boxes indicate the metabolites that were significantly increased. Bar charts show raw intensity at respective time points (1 and 4 h). Bars labelled with an asterisk indicate significant changes in the abundance of metabolites (q < 0.05; ≥1 − log_2_FC). Raw intensity was normalized relative to the median, log_2_ transformed, and auto-scaled. Data represent geometric means of normalized intensity ± standard deviation (*n* = 3).

Cell envelope biogenesis was significantly perturbed following treatments with the polymyxin B–enrofloxacin combination (**Figure 7**). The level of UDP-*N*-acetyl-d-glucosamine (log_2_FC = −2.07) was decreased at 1 h in response to polymyxin B alone but increased at 4 h following treatment with the combination (log_2_FC = 2.45). Interestingly, a similar trend was also observed for UDP-*N*-acetylmuramoyl-l-alanyl-d-glutamyl-6-carboxyl-lysyl-d-alanyl-d-alanine at 4 h in response to the combination (log_2_FC = 1.45), whereas enrofloxacin alone had minimal effects on the cell envelope biogenesis pathway at either time point (**Figure 7**).

## Discussion

Resistance to the last-resort polymyxins can emerge after monotherapy (Bergen et al., 2010; Abdul Rahim et al., 2015; Schneider et al., 2016; Tran et al., 2016b); therefore, rational polymyxin combinations with other antibiotics have been strongly recommended from the PK/PD perspective (Nation et al., 2015). The use of polymyxin B in combination with enrofloxacin, a fluoroquinolone, is highly effective against polymyxin- and enrofloxacin-resistant XDR *Pseudomonas aeruginosa* (Lin et al., 2018). To our knowledge, the present study is the first to demonstrate that the synergistic killing of polymyxin B in combination with enrofloxacin was time dependent, which was initially facilitated by polymyxin B and subsequently driven by enrofloxacin.

Enrofloxacin is partially metabolized into ciprofloxacin *in vivo* by the cytochrome P450 enzymes (Küng et al., 1993; Kaartinen et al., 1995; Giguere et al., 1996; Mengozzi et al., 1996; Salvadori et al., 2015), and its PK profile in humans is currently undetermined; therefore, clinically achievable concentrations of ciprofloxacin were used for enrofloxacin (1 mg/L) in the present study (Sánchez Navarro et al., 2002). Polymyxin B was examined at 2 mg/L to ensure the clinical relevance of our findings (Sandri et al., 2013). With an inoculum of 10^8^ CFU/ml, neither 2 mg/L of polymyxin B nor 1 mg/L of enrofloxacin had a significant killing effect on XDR *P. aeruginosa* 12196; surprisingly, the combination of polymyxin B and enrofloxacin exhibited synergistic killing at 4 h. In order to understand the molecular basis of the dynamic extensive killing, bacterial metabolic profiles were examined at 1 and 4 h following antibiotic treatment (**Figure 1**).

Consistent with the phenotypical synergistic killing observed in our previous PK/PD studies, our metabolomics results showed that the combination was clearly separated from each monotherapy alone at both time points (**Figure 1**). Metabolic pathway analyses revealed that the synergistic killing of polymyxin B–enrofloxacin combination was attributed to the perturbations of key metabolic pathways, including lipid, carbohydrate, nucleotide, and energy metabolism (**Figures 1**, **2** and **S2**; **Supplementary Tables 1–2**). A large number of metabolites associated with fatty acids and lipids were significantly perturbed following polymyxin B alone and the combination at 1 and 4 h (**Figure 3**). These findings are in agreement with the primary mode of action of polymyxins *via* the disruption of the bacterial outer membrane (Li et al., 2006). Notably, this finding is also consistent with previous results in *Acinetobacter baumannii*, in which colistin significantly perturbed the gene expression and metabolites in fatty acid and lipid metabolism (Henry et al., 2015; Maifiah et al., 2017). On the contrary, enrofloxacin alone did not affect fatty acid and lipid metabolisms at 1 and 4 h (**Figures 1**, **3** and **S2**), which is consistent with its mode of action by the inhibition of topoisomerase II (DNA gyrase) and prevention of the replication of DNA (Küng et al., 1993; Kaartinen et al., 1995; Giguere et al., 1996; Mengozzi et al., 1996; Salvadori et al., 2015). DNA damage activates the SOS gene network that results in the production of DNA repair proteins (Power and Phillips, 1992) and the accumulation of nucleotides (Dörries et al., 2014). Furthermore, transcriptomic analysis of *Staphylococcus aureus* showed that fluoroquinolone treatment up-regulates the expression of ribonucleotide reductases and several genes involved in the DNA repair pathways (Cirz et al., 2007). This finding is consistent with the metabolomics data obtained in the present study with *P. aeruginosa*. Our results showed that SOS responses induced by enrofloxacin alone and the combination resulted in the accumulation of pyrimidine metabolites to cope with the inhibition of DNA replication at 4 h (**Figure 4**). Consistently, the level of d-ribose 5-phosphate in PPP, a key precursor in nucleotide metabolism, was also significantly increased following the treatment with the combination at 4 h, but not at 1 h when polymyxin killing predominated (**Figure 6**). Overall, our metabolomics results highlight the dominant effect of enrofloxacin on the synergy observed with the combination at 4 h.

Despite the largely overlapping metabolic perturbations between polymyxin B or enrofloxacin alone and the combination, the present data revealed that the synergistic combination induced several unique metabolic alterations in energy metabolism (**Figures 2** and **5**) and cell envelope biogenesis (**Figure 7**). It is evident that the combination uniquely caused a significant depletion of key redox co-factors, including FMN and NADPH, at 1- and 4-h posttreatment (**Figure 5**). It is likely that *P. aeruginosa* diverted the energy required to synthesize nucleotides toward DNA repair, as a result of the activation of the SOS gene network. The observed decrease in energy metabolism coupled with significant perturbations in PPP suggests an imbalanced redox state due to the treatment with this combination. Interestingly, at 4 h, the combination led to significant increase in fatty acids levels and decrease in phospholipid levels (**Figure 3**). These alterations might be attributed to the reduced utilization as an energy source and membrane remodelling. Overall, our findings indicate that the inhibition of energy metabolism plays a key role in the mechanism of synergistic bacterial killing by the combination at 4 h.

Moreover, the combination displayed significant and persistent effects on the cell envelope biogenesis in XDR *P. aeruginosa* (**Figure 7**). At 4 h, a significant increase in the levels of a peptidoglycan biosynthesis metabolite UDP-*N*-acetylmuramoyl-l-alanyl-d-glutamyl-6-carboxy-l-lysyl-d-alanyl-d-alanine was observed with the combination (**Figure 7**). In *Streptococcus faecalis*, the inhibition of DNA replication by a fluoroquinolone resulted in the formation of thicker cell wall (Higgins et al., 1974). It is very likely that the synergistic killing at 4 h by the combination is driven by the secondary antibiotic, enrofloxacin. In addition to the inhibition of cell wall biogenesis, the combination synergistically inhibited the lipopolysaccharide (LPS) modification pathway (**Figure 2**). Polymyxin B alone against *P. aeruginosa* leads to the development of resistance most commonly *via* lipid A modification with aminoarabinose (Miller et al., 2011). UDP-l-Ara4FN, a key precursor of lipid A modification (Breazeale et al., 2005; Gatzeva-Topalova et al., 2005), was significantly enriched following the treatment with polymyxin B alone at both 1 and 4 h; however, this effect was not observed with the combination (**Figure 2**). Our results clearly demonstrated that polymyxin resistance *via* the lipid A modification can emerge rapidly (e.g., as early as in 1 h) even in resistant isolates. Importantly, the inhibition of polymyxin resistance by enrofloxacin plays a key role in the synergistic antibacterial killing. From a PK/PD perspective, our metabolomics results are clinically significant and highlight the importance of a combination therapy in minimizing the development of resistance to the last-line polymyxins.

## Conclusions

The development of effective polymyxin combination therapy is of utmost importance in response to the increasing incidence of infections caused by XDR Gram-negative “superbugs.” To the best of our knowledge, this is the first systems pharmacology study to investigate the synergistic effect of polymyxins with a fluoroquinolone antibiotic against XDR *P. aeruginosa*, which is resistant to all antibiotics, including polymyxins and fluoroquinolones. Importantly, co-administration of enrofloxacin reduced the emergence of polymyxin resistance by inhibiting lipid A modification. These results provide important mechanistic insights into optimizing the clinical use of this promising combination using PK/PD approaches.

## Author’s Note

This article is dedicated to the memory of Professor Alan Forrest, a friend of many and an inspiring researcher.

## Data Availability Statement

All datasets generated for this study are included in the article/**Supplementary Files**.

## Author Contributions

JL and TV conceived the project, and all authors were involved in the design of the experiments. Y-WL and M-LH performed the experiments, and Y-WL, M-LH, JZ, YZ, GR, AF, JS, KK, PH, AP, DC, and QZ analyzed the results. All authors reviewed the manuscript.

## Funding

JL, TV, GR, AF, JS, PH, AP, DC, and KK are supported by a research grant from the National Institute of Allergy and Infectious Diseases of the National Institutes of Health (R01 AI111965). Y-WL and M-LH are recipients of the 2018–2019 Faculty Bridging Fellowship, Monash University. GR, KK, QZ, TV and JL are supported by the National Institute of Allergy and Infectious Diseases of the National Institutes of Health under Award Numbers R01AI132681 and R01AI146160.

## Conflict of Interest

The authors declare that the research was conducted in the absence of any commercial or financial relationships that could be construed as a potential conflict of interest.
