# Supplementary material for: Synergistic Combination of Polymyxin B and Enrofloxacin Induced Metabolic Perturbations in Extensive Drug-Resistant Pseudomonas aeruginosa
Source: Front Pharmacol. 2019 Oct 3;10:1146. doi: 10.3389/fphar.2019.01146 (PMC6785843; doi:10.3389/fphar.2019.01146)
Supplement: Supplementary file 2 [file DataSheet_2.docx]

**Synergistic Combination of Polymyxin B and Enrofloxacin Induced Metabolic Perturbations in Extensive Drug-resistant *Pseudomonas aeruginosa***

Yu-Wei Lin^1#^, Mei-Ling Han^1#^, Jinxin Zhao^1^, Yan Zhu^1^, Gauri Rao^2^, Alan Forrest^2†^, Jiangning Song^3^, Keith S Kaye^4^, Paul Hertzog^5,6^, Anthony Purcell^3^, Darren Creek^7^, Qi Tony Zhou^8^, Tony Velkov^9^*, and Jian Li^1*^

^1^Monash Biomedicine Discovery Institute, Infection and Immunity Program and Department of Microbiology, Monash University, Clayton, Victoria 3800, Australia. ^2^Division of Pharmacotherapy and Experimental Therapeutics, Eshelman School of Pharmacy, University of North Carolina, Chapel Hill, NC 27599, USA. ^3^Monash Biomedicine Discovery Institute, Infection and Immunity Program and Department of Biochemistry and Molecular Biology, Monash University, Clayton, Victoria 3800, Australia. ^4^Department of Medicine, Division of Infectious Diseases, Detroit Medical Center, Wayne State University, 3990 John R, Detroit, MI 48201, USA. ^5^Centre for Innate Immunity and Infectious Diseases, Hudson Institute of Medical Research, Clayton, Victoria 3168, Australia. ^6^Department of Molecular and Translational Sciences, School of Clinical Sciences at Monash Health, Monash University, Clayton, Victoria 3168, Australia. ^7^Drug Delivery, Disposition and Dynamics, Monash Institute of Pharmaceutical Sciences, Monash University (Parkville campus), Parkville, Victoria 3052, Australia. ^8^Department of Industrial and Physical Pharmacy, College of Pharmacy, Purdue University, West Lafayette, Indiana 47907, USA. ^9^Department of Pharmacology and Therapeutics, The University of Melbourne, Melbourne, Victoria 3800, Australia.

*Corresponding author: Jian Li, telephone: +61 3 9903 9702, fax: +61 3 9905 6450, Email: jian.li@monash.edu.


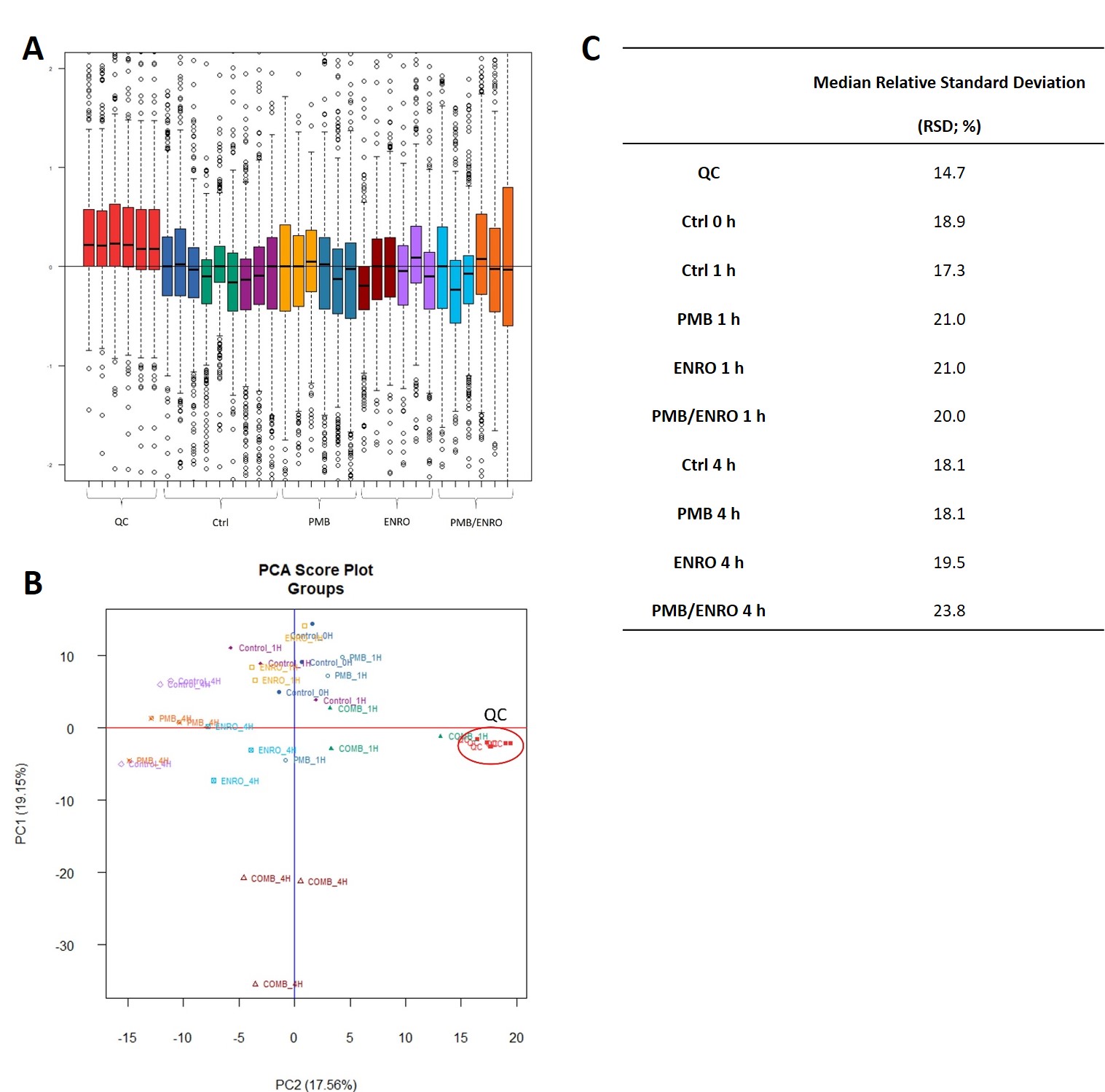


**Figure S1.** Data precision of different groups for all assessed intracellular metabolites. **(A)** Log-transformed relative abundance of each treatment group; **(B)** principal component score plot for the metabolites from all groups; and **(C)** median relative standard deviation (% RSD) for each group. QC = quality control; Ctrl = control; PMB = polymyxin B; ENRO = enrofloxacin; PMB/ENRO = polymyxin B + enrofloxacin.


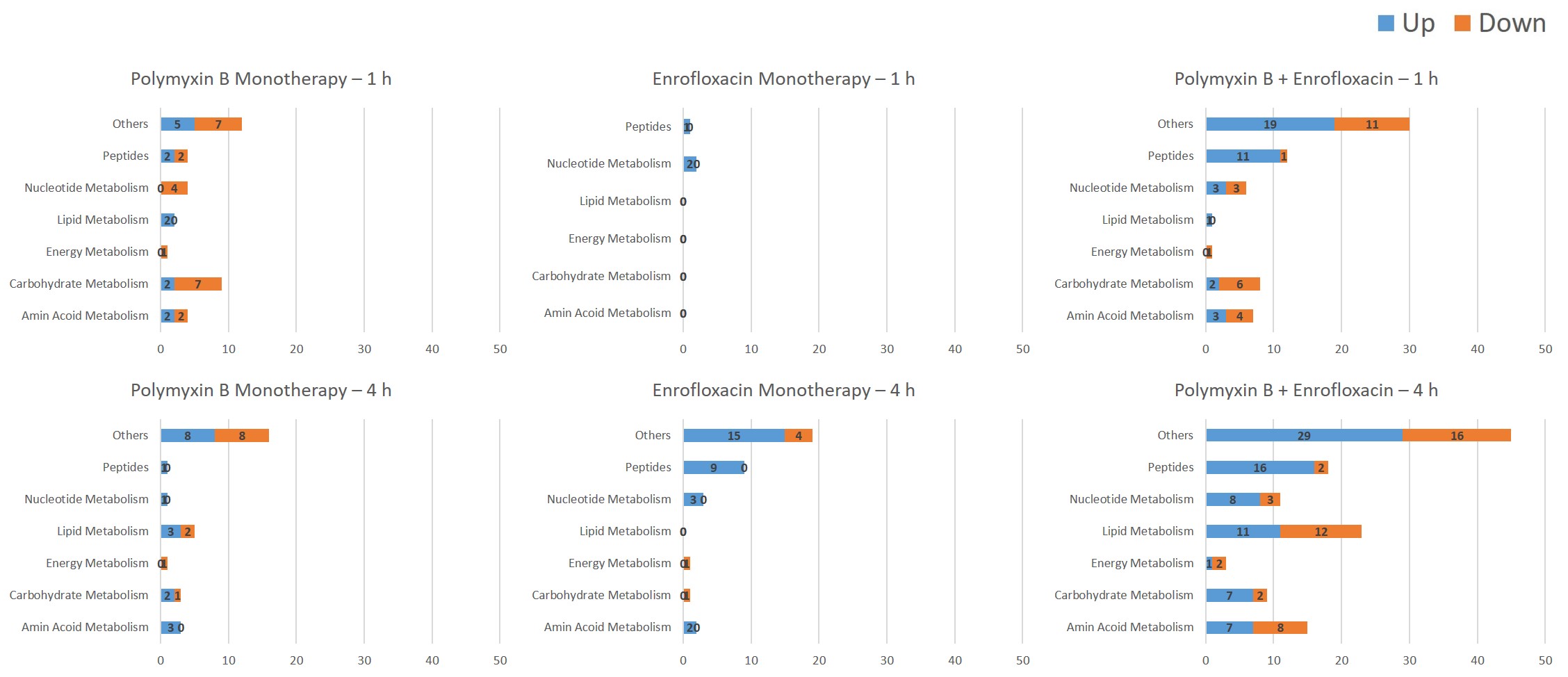


**Figure S2.** Numbers of significantly perturbed metabolites following treatments with polymyxin B and enrofloxacin alone and in combination against extensive drug-resistant (XDR) *Pseudomonas aeruginosa* 12196. The class designated as Others includes cofactors and vitamins, glycan, secondary metabolites and undefined metabolites.
